# Effects of Particulate Air Pollution on Cardiovascular Health: A Population Health Risk Assessment

**DOI:** 10.1371/journal.pone.0033385

**Published:** 2012-03-14

**Authors:** Jing Feng, Wei Yang

**Affiliations:** School of Community Health Sciences, University of Nevada, Reno, Nevada, United States of America; National University of Singapore, Singapore

## Abstract

Particulate matter (PM) air pollution is increasingly recognized as an important and modifiable risk factor for adverse health outcomes including cardiovascular disease (CVD). However, there are still gaps regarding large population risk assessment. Results from the nationwide Behavioral Risk Factor Surveillance System (BRFSS) were used along with air quality monitoring measurements to implement a systematic evaluation of PM-related CVD risks at the national and regional scales. CVD status and individual-level risk factors were collected from more than 500,000 BRFSS respondents across 2,231 contiguous U.S. counties for 2007 and 2009. Chronic exposures to PM pollutants were estimated with spatial modeling from measurement data. CVD outcomes attributable to PM pollutants were assessed by mixed-effects logistic regression and latent class regression (LCR), with adjustment for multicausality. There were positive associations between CVD and PM after accounting for competing risk factors: the multivariable-adjusted odds for the multiplicity of CVD outcomes increased by 1.32 (95% confidence interval: 1.23–1.43) and 1.15 (1.07–1.22) times per 10 µg/m^3^ increase in PM_2.5_ and PM_10_ respectively in the LCR analyses. After controlling for spatial confounding, there were moderate estimated effects of PM exposure on multiple cardiovascular manifestations. These results suggest that chronic exposures to ambient particulates are important environmental risk factors for cardiovascular morbidity.

## Introduction

The deleterious effects of air pollution on cardiac function have been well documented in animal studies: acute exposure to particulate air pollutants has been linked to ischemia-reperfusion injury [1]–[2], while long-term exposure has been demonstrated to augment the development of atherosclerosis [3]–[4]. A potential relation between air pollution and cardiovascular ill health has also been described in humans: a wide variety of time-series analyses have associated recent exposure to pollution episodes with increases in morbidity and mortality related to cardiovascular complications [5]–[Bibr pone.0033385-Morris1] [Bibr pone.0033385-Goldberg1] [Bibr pone.0033385-Peters1] [Bibr pone.0033385-Pope1] [Bibr pone.0033385-Pope2] [Bibr pone.0033385-Samet1] [Bibr pone.0033385-Schwartz1] [Bibr pone.0033385-Schwartz2] [14]; further, cohort studies such as the Harvard Six Cities Study and the American Cancer Society (ACS) Cancer Prevention Study II, have reported significant associations between long-term exposure to particulate matter (PM) air pollution and increased all-cause and cardio-respiratory mortality [15],[16]. The increased mortality risk was confirmed in extensive reanalyses and new analyses providing compelling evidence for a potential role of elevated PM concentrations in cardiovascular injury [17]–[Bibr pone.0033385-Miller1] [Bibr pone.0033385-Zeger1] [20].

To extend previous analyses primarily concerned with cardiovascular deaths and hospitalization, this paper attempts to evaluate the long-term relationship between prevalent CVD and PM across the general population in the United States. In particular, lifestyle factors, socioeconomic attributes and comorbid conditions that are major CVD risk factors were considered together with ecologic air quality covariates to provide a broad context of risk assessment. Although epidemiologic studies are not geared to definitive analyses of the biological pathways from exposure to response, they can provide empirical evidence to help evaluate plausible biological explanations, and thus enhance our understanding of the long-term cardiovascular health effects of PM pollution.

## Methods

### Individual-level data

Data on CVD status and individual covariates were obtained from the U.S. Behavioral Risk Factor Surveillance System (BRFSS), a random digit-dialing cross-sectional household survey system which began to monitor CVD status among U.S. adults (18+ years old and non-institutionalized) since 2005. Developed by the Centers for Disease Control and Prevention, the BRFSS is the largest telephone health survey in the world and currently collects information on preventive health practices and risk behaviors as well as a wide range of health outcomes in 50 states, the District of Columbia (DC), and three territories. Participants were selected using probability sampling from all households with telephones in each state or territory at 1^st^ stage and all adults per household at 2^nd^ stage (1 adult selected per household) [21]–[Bibr pone.0033385-Nelson1] [23]. The survey questions pertaining to CVD were threefold, “Has a doctor, nurse, or other health professional ever told you that you had any of the following? (1) A heart attack, also called myocardial infarction (MI); (2) angina or coronary heart disease (CHD); and (3) stroke (STK).” Since data on relevant medical conditions (e.g. hypertension, high cholesterol) were only collected in odd-numbered years and to maintain coherence in survey protocols used to ascertain CVD status, the analysis was restricted to the BRFSS of 2007 (No. of valid responses [N-VR] = 430,912; median interview completion rate among participating states [MICR-PS] = 75.2%) and 2009 (N-VR  = 432,607; MICR-PS = 77.6%). Most of the respondents were non-Hispanic white (>85%), resulting in inadequate racial gradients and data sparseness after full adjustment for variations in race/ethnicity (the proportion of minority respondents of Asian, Native Hawaiian/ Pacific Islander, American Indian/Alaska Native, and/or multiracial origins was <5%). Study samples were thus restricted to non-Hispanic whites, African Americans and Hispanics to control data dimensionality and to accommodate potential biases arising from racial/ethnic distributions which may covary with the exposure of interest. The BRFSS data were deidentified and thus analyzed anonymously (all survey instruments and data are available at www.cdc.gov/brfss).

### Ambient air pollution data

Concentration data available for estimating background levels of PM_10_ (coarse/fine particle with diameter <10 µm) and PM_2.5_ (fine particle with diameter <2.5 µm) were extracted from the U.S. Environmental Protection Agency's Air Quality System [24]. Measurements from years 1999–2005 were obtained for PM_10_ and PM_2.5_ (no systematic sampling of PM_2.5_ before 1999); for exposure assessment, data were further restricted to surveillance-type monitoring stations located throughout urban and rural areas in conterminous states, and to those with at least 50% of observations (percent of observations calculated as the ratio of valid days to scheduled days for the year). Yearly median value was the chosen measure of background particulate concentration. They were constructed from site-level PM concentrations computed as integrated averages of hourly samples collected in a 24-hour period. Table 1 shows the distributions of PM concentrations based on data from the selected period and monitoring sites. As a means of quantifying year round exposure across region, median PM_10_ and PM_2.5_ concentrations obtained from the sampling sites had fairly strong correlations for the study period (Table 1).

**Table 1:** Distributions of PM_10_ and PM_2.5_,1999–2005, surveillance-oriented sites from contiguous U.S. region.

Pollutant^a^	Study	Median sampling days^c^		Yearly median levels (µg/m^3^)					
	sites^b^	Per	Period	Mean	25^th^	50^th^	75^th^	100^th^	Inter-quartile
		year	total	(SD)	percentile			percentile	range
PM_10_	853	60	384	19.7 (1.4)	17.0	20.2	23.9	59.7	6.9
PM_2.5_	734	112	656	10.7 (1.4)	8.9	11.6	13.4	21.9	4.6
Average correlation of yearly site-specific median measurements: PM_10_ — 0.86 PM_2.5_ — 0.81									

a Following the promulgation of the National Ambient Air Quality Standard for PM_2.5_ in 1997, routine collection of PM_2.5_ was implemented in 1999. No attempt was made to convert PM_10_ concentrations to PM_2.5_, which requires a scaling factor based on a presumptive proportion of PM_2.5_ in the PM_10_ mass.

b Sites describe unique sampling points indicated by longitude/latitude. Those providing no geodetic datum information were not included.

c While PM was typically measured at a frequency of every six days or higher, many sites took daily sampling.

### Geostatistical methods

Population exposures to PM at the available areal level were assessed on the basis of long-term averaged yearly median concentrations by kriging in conjunction with block interpolation. County was the target interpolation block as the nationwide BRFSS does not record individuals' residence at the city or town level currently. The kriging technique quantifies spatial dependence represented by available observations, and uses the estimated autocorrelation structure to form minimum variance estimators over the entire study domain [25],[26]. For this study, all sample points representing surveillance-type monitoring sites were used in model development for accurate spatial interpolation. Particulate concentrations were transformed to a logarithmic scale to better approximate a normal residual distribution and to constrain the modeled concentrations to be positive. Sampled data were first checked for autocorrelation and trends so as to determine the optimal parameters that characterize difference-squared values between each pairs of points at different distances lags (i.e. semivariogram); multiple semivariogram models were then fitted with different specifications on distance lags and directional influences. The optimal kriging parameters were chosen based on leave-one-out cross-validated error statistics including the mean prediction error (ME), root-mean-squared-error (RMSE) and cross-validated R^2^. A 44×44 km grid partition was used to convert point-kriged results to raster coverages at the continental scale. Area-based exposure assignment was subsequently made by computing block averages over discretized surfaces. All geostatistical analyses were implemented with ESRI ArcGIS (v9.3; ESRI Inc., Redlands, CA, USA).

### Statistical analysis

**Figure 1:**
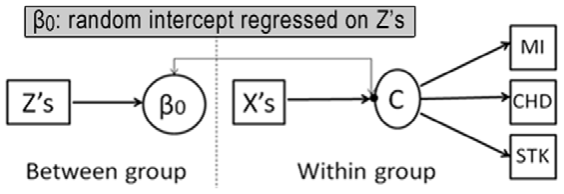
A schematic depiction of the multilevel latent class regression model.

Risks for individual CVD components (i.e. MI, CHD, STK) were estimated by standard mixed-effects logistic regression using the multilevel pseudo-maximum likelihood (MPML) method; MPML estimates of the overall CVD risks were obtained with multilevel latent class regression (LCR) [27]–[Bibr pone.0033385-Formann1] [Bibr pone.0033385-Clogg1] [30]. This modeling approach posits that individuals form homogenous classes based on discrete observed variables (e.g. self-reports of CVD status), and class membership depends on a latent construct that serves as a summary of observed indicators. For the multilevel LCR analysis, a two-class or binary latent construct (denoted by C) was hypothesized (high vs. low CVD risks), with three categorical indicators obtained as item responses to the BRFSS CVD module enquiring the occurrence of MI, CHD, and STK. Class membership was characterized by both individual and group-level risk factors (denoted by X's and Z's respectively) for CVD, including age, gender, race, income, education, hypertension, hypercholesterolemia, diabetes, smoking, physical activity level, obesity, and ambient concentrations of PM spatially interpolated to each county. The multilevel LCR model is schematically depicted in Figure 1.

The conceptual equivalence between a latent class and random effects specifications has been demonstrated previously [31]–[Bibr pone.0033385-Vermunt1] [33]. Per standard mixed-effects modeling, a random intercept deviation (for each county) was adopted to represent a covariance structure induced by county-to-county heterogeneity (i.e. interdependencies of individual observations within each county). The additional latent class specification is beneficial for synthesizing correlated item responses into an outcome that is easily interpretable while retaining latent heterogeneity in the data (i.e. unobserved but not strictly exogenous differences between individuals) as a means of controlling for unobserved influences that contribute to the relations between observed variables. Because there were empirical associations among the responses of interest (the three CVD indicators had a Fleiss Kappa coefficient of 0.3, implying a fair degree of agreement), and analyses stratified by CVD indicators unnecessarily attenuated the statistical relationship between them, the latent outcome formulation provided an improvement on bias control as it took into account the interdependency of reported cardiovascular symptoms in estimating the standard errors used in hypotheses testing.

The probability for a cardiovascular response in the form of MI, CHD or STK, or being a member of a high-risk class is defined in terms of binomial logit. Because of evidence of a curvilinear relationship between BMI and cardiovascular outcomes, linear and squared terms for BMI were used as continuous variables in model fitting. To allow for broad-scale regional effects, indicator variables representing the nine Census regions were employed; analyses were also stratified by the nine Census regions to explore the possibility of effect modification by region. Model evaluation consisted primarily of comparing hierarchically consistent candidate models on likelihood-based information criteria. Statistical programs SAS (v9.2; SAS Institute Inc., Cary, NC, USA) and Mplus (v6.1; Muthén & Muthén, Los Angeles, CA, USA) were used for the mixed-effects analyses.

## Results

### Spatial variations in background PM concentrations

Ordinary and universal kriging procedures were evaluated as methods to estimate the long-term averaged median PM concentrations. Table 2 summarizes the performance metrics for the preferred models with and without a spatial trend component. The models gave similar overall performance measures: incorporating a linear or quadratic trend component did not give a stronger basis for interpolation (as indicated by RMSE values).

**Table 2:** Evaluation statistics (µg/m^3^) for the exposure assessment methods.

Pollutant	Yearly median levels				Kriging method (log transformed data)								
	Observed		Estimated		Ordinary^b^			Universal			Universal		
								linear trend			quadratic trend		
	Mean	SD	Mean	SD	ME	RMSE	R^2^	ME	RMSE	R^2^	ME	RMSE	R^2^
PM_10_ (N^a^ = 853)	19.663	1.430	19.716	1.269	0.0027	0.264^c^	0.453	−0.0074	0.276	0.404	0.0021	0.271	0.425
PM_2.5_ (N = 734)	10.664	1.376	10.339	1.316	0.0021	0.165^c^	0.734	−0.0016	0.165	0.733	−0.0032	0.171	0.714

a N is the number of surveillance-oriented sites used for PM pollution modeling.

b A constant trend is implied by ordinary kriging.

c The optimal assessment method is indicated. When models rank similarly in terms of performance, the simpler specification that reproduces important features of the empirical variogram is deemed optimal.

**Figure 2:**
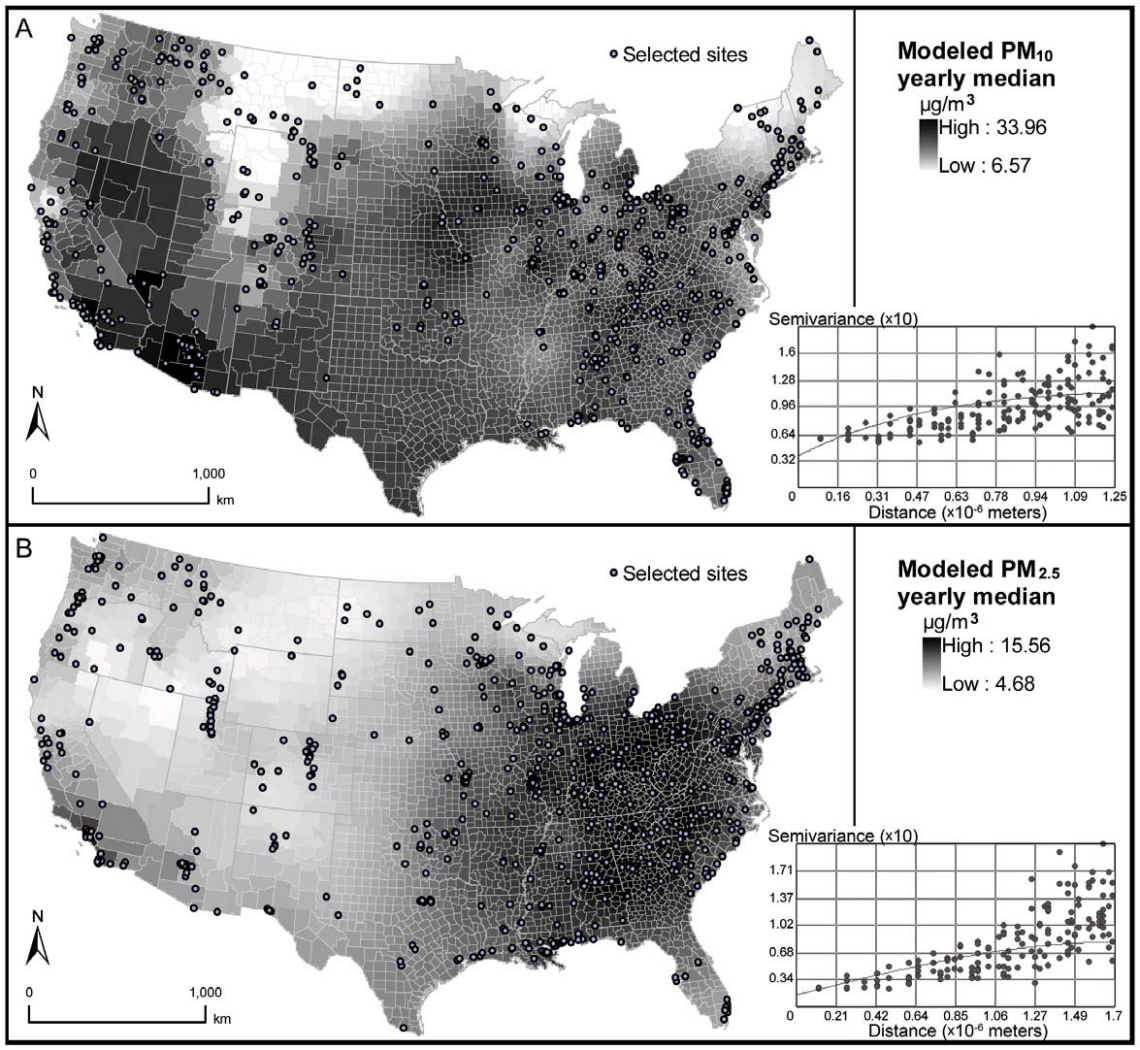
Estimated background PM_10_ and PM_2.5_ concentrations (µg/m^3^) across contiguous U.S. counties. A—PM_10_ yearly median concentrations (averaging 1999–2005), assessed with ordinary kriging, exponential covariance, lag distance = 125 km, nugget = 0.037, range = 1,538 km, partial sill = 0.083; B—PM_2.5_ yearly median concentrations (averaging 1999–2005), assessed with ordinary kriging, spherical covariance, lag distance = 170 km, nugget = 0.014, range = 1,687 km, partial sill = 0.066.

Exposure estimation results showed that the chosen kriging models did not extrapolate much beyond the range of measured concentrations; however, estimated values have a markedly lower standard deviation (Table 2). Such discrepancies possibly arose from monitor placement bias as they tend to lie in urban, more polluted areas, whereas the modeled concentrations utilized measurements from neighboring samples to provide full coverages across measurement units. As such, they may give “smoothed” spatial patterns of pollution levels and underestimate exposure gradients. Figure 2 shows the median background PM concentrations across contiguous U.S. counties for the selected time window, based on the optimal modeling methods (defined as those with the lowest RMSE values). Interpolated PM surfaces were similar for the preferred kriging models, as indicated by the high correlations (>0.95) between estimates assessed with the different models. This suggests that the background PM pollution landscape for the study region and time frame was unlikely to change greatly depending on the choice of the optimal spatial interpolation model. Figure S1 in the Supporting Information provides graphical comparisons between measured concentrations at the study sites and predicted values by the chosen kriging methods.

### Cardiovascular risk estimation

**Figure 3:**
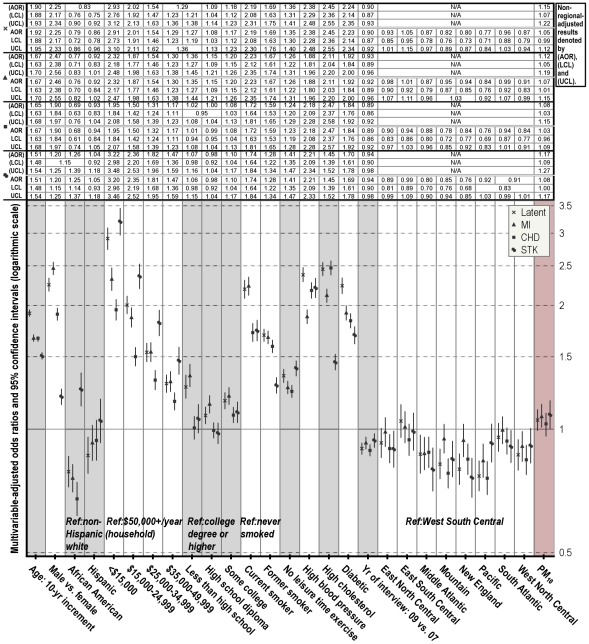
Multivariable-adjusted odds ratios (AOR) and lower and upper 95% confidence limits (LCL & UCL) for CVD complications from PM_10_-fitted models —assessed with samples from the 2007 and 2009 Behavioral Risk Factor Surveillance System. Both regionally and non-regionally adjusted results were presented, with the former graphically displayed. BMI and (BMI-squared)/100 were included as continuous variables. PM_10_-related effects were associated with 10 µg/m^3^ increment in yearly median levels.

**Figure 4:**
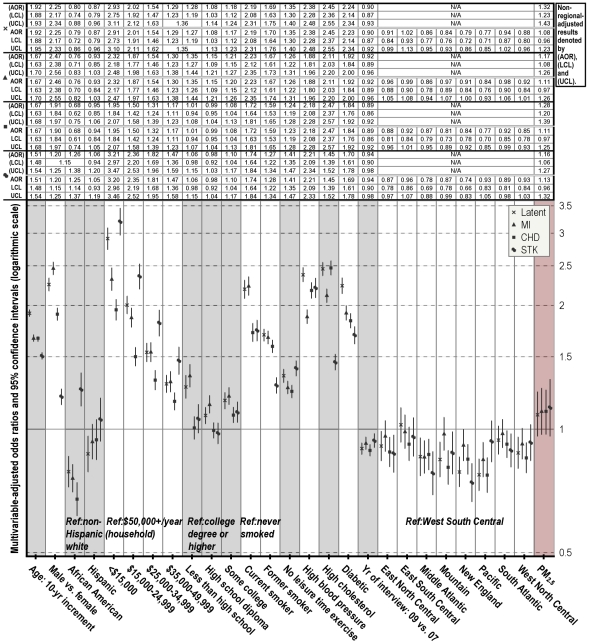
Multivariable-adjusted odds ratios (AOR) and lower and upper 95% confidence limits (LCL & UCL) for CVD complications from PM_2.5_-fitted models —assessed with samples from the 2007 and 2009 Behavioral Risk Factor Surveillance System. Both regionally and non-regionally adjusted results were presented, with the former graphically displayed. BMI and (BMI-squared)/100 were included as continuous variables. PM_2.5_-related effects were associated with 10 µg/m^3^ increment in yearly median levels.

For the assessment of cardiovascular health in relation to individual and ecologic co-risk factors, the 2007 and 2009 BRFSS data were linked to the estimated background PM concentrations by county of residence. Covariate missingness was analyzed with non-response indicators constructed for items on which missing data may not occur randomly (e.g. income and education). Because of little evidence for associations of missingness indicators with CVD, the final study populations included only survey respondents with known responses on all individual covariates, and were limited to those residing in the 48 conterminous states and DC, of which 2,231 counties participated in the 2007 and 2009 BRFSS cardiovascular health survey module. The size of the samples ranged from 494,358 to 499,667, depending on the specific CVD components assessed separately or in combination as the outcome measure (taken together, a total of 500,715 responses were evaluated). The samples were approximately 39% men and 61% women, and the median age of participants 56 years. The age and sex characteristics of study subjects were comparable across levels of PM exposure (Table S1); the racial, socioeconomic and lifestyle traits were distributed somewhat unevenly across PM pollution ranges. The crude prevalence estimates were 6.2% (31,078) for MI, 6.6% (32,752) for CHD, and 3.9% (19,589) for STK. Estimated posterior probabilities indicated fairly homogeneous overall latent class patterns across all fitted models: around 11% of respondents constituted the high-risk class. Age, sex and race-adjusted prevalence was estimated for MI, CHD and STK (at mean values of individual-level covariates for the study population) in model building, with random effects adjustment of county-specific deviations in CVD outcomes. Overall, higher CVD rates occurred in the South and Midwest than in the Northeast and West (Figure S2); and higher-than-average particle concentrations occurred in the southern-central region (Table S2). Figures 3 and 4 show the PM pollution effect estimates from the final fitted models controlling for competing risk factors, with adjustment for spatial and temporal trends in disease. All individual-level covariates were independently associated with CVD outcomes, and their effect estimates showed little change across models.

**Figure 5:**
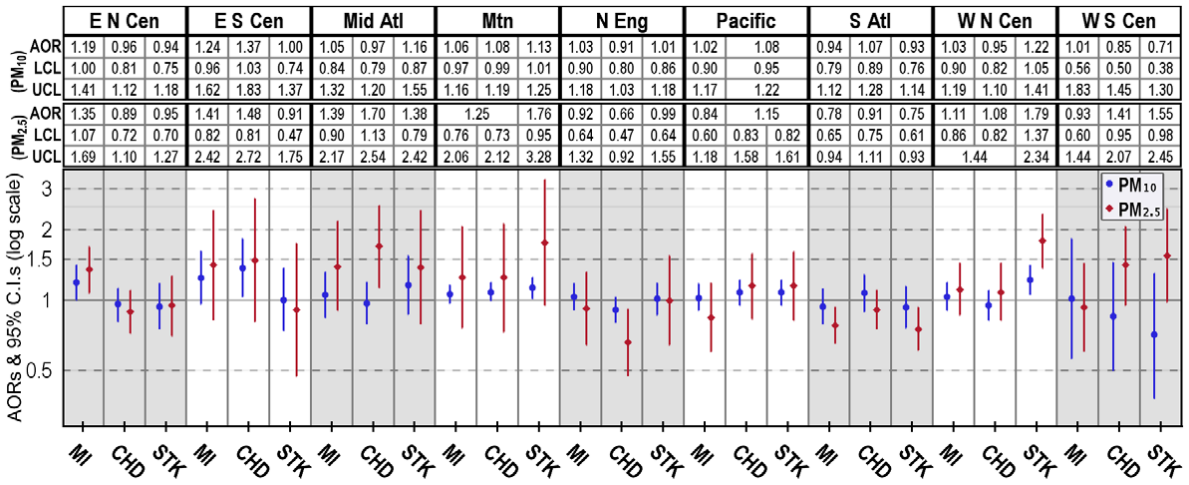
Region-specific multivariable-adjusted odds ratios (AOR) and lower and upper 95% confidence limits (LCL & UCL) per 10 µg/m^3^ increment in PM for CVD complications, controlling for age, gender, race, education, income, smoking status, physical activeness, BMI (linear and quadratic terms), hypertension, hypercholesteraemia, diabetes, and year of interview.

PM_10_ or PM_2.5_ alone were associated with MI, CHD and STK after accounting for effects attributable to age, sex, race, education, income, BMI, hypertension, hypercholesterolemia, diabetes, smoking status, physical activeness, and temporal patterns in CVD (year of interview used as the time index) (Figures 3 and 4). The multivariable-adjusted odds ratio (AOR) for MI was estimated at 1.12 (95% CI: 1.05–1.19), for CHD 1.08 (1.03–1.15), for STK 1.17 (1.09–1.27), and for overall susceptibility 1.15 (1.07–1.22) per 10 µg/m^3^ increase in yearly PM_10_ median concentrations. PM_2.5_ showed slightly stronger effects on overall cardiovascular morbidity, with an estimated AOR for MI of 1.17 (1.08–1.26), for CHD 1.28 (1.20–1.39), for STK 1.16 (1.06–1.27), and for overall susceptibility 1.32 (1.23–1.43) per 10 µg/m^3^ increase in yearly median concentrations. However, inclusion of geographic location indicators attenuated the PM-CVD associations, with significant effects only observed on MI (AOR = 1.07; 95% CI: 1.01–1.15) and STK (1.08; 1.00–1.17) from PM_10_ exposure, whereas CVD risks associated with PM_2.5_ exposure remained elevated, if not significant. On considering possible effect modification by spatial location, region-specific models controlling for individual and temporal covariates were also assessed. Although the region-stratified approach does not provide a test of statistical significance of the differences between the stratified odds ratios, there was a mild indication of a region-PM interaction (Figure 5): PM showed strongest associations with MI in ENCen, with CHD in ESCen and Mid Atl, and with STK in WNCen; the inverse PM associations with MI and STK estimated from SAtl and with CHD from NEng regions were likely due to discordance between morbidity and background PM across Central Florida and Maine counties respectively (Figure 2 and Figure S2).

## Discussion

This study used time-averaged ambient air pollution data and a cross-sectional sample of 500,715 adults to assess CVD risks associated with background PM pollution across contiguous U.S.. There have been only a few previous studies that assessed long-term air pollution effects on CVD across large populations, partly due to the lack of direct exposure measurements at a broad scale. To address this limitation, reasonable spatial interpolation models were developed to enable population exposure assessment. On the basis of multilayered data, PM effects were evaluated on cardiovascular complications while directly adjusting for individual differences in major risk factors. There were moderate estimated effects of PM exposure on cardiovascular morbidity: multivariable-adjusted odds for the multiplicity of CVD outcomes increased by 1.32 and 1.15 times per 10 µg/m^3^ increase in PM_2.5_ and PM_10_ respectively in the LCR analyses; the estimated PM_2.5_ effects diminished quite a bit following spatial adjustment with indicators distinguishing the nine Census regions, while the spatially adjusted PM_10_ effects on MI and STK remained marginally significant (Figures 3 and 4). The effects of PM_10_ cannot be independently quantified from those of PM_2.5_ based on available data granularity however, since PM_2.5_ is a key component of the total PM_10_ mass.

Although differences in study design, endpoint/exposure assessment, and population or region covered limit the scope for direct comparison with earlier PM-related mortality studies, the moderate relative CVD risks associated with PM exposure found in the current study were roughly in line with previous findings. In the Six Cities Study, Dockery et al. estimated an adjusted cardiopulmonary mortality rate ratio of 1.26 for the most polluted versus the least polluted city using fine particles as measures of pollution [15]. In the ACS Study carried out by Pope et al., the adjusted relative mortality risks from cardiopulmonary causes were 1.26 and 1.31 times higher in the most polluted areas as in the least polluted in terms of sulfates and fine particles levels respectively [16]. A stronger correlation between fine particle pollution and cardiovascular mortality was found by Miller et al. in their Women's Health Initiative observational study; they estimated a hazard ratio of 1.76 for death from CVD per 10 µg/m^3^ increase in the mean PM_2.5_ concentration [18]. In the reanalysis of the Six Cities Study and ACS Study data by the Health Effects Institute (HEI), inclusion of auxiliary socio-demographic and environmental variables at the areal level was shown to have little impact on the estimated associations between particulate pollution and cardiopulmonary mortality; however, risk estimates were somewhat sensitive to adjustment for spatial patterns in the ACS Study data [17]. This may reflect a spatial trend of disease burden which likely contributes to the observed PM-CVD relationship. In their Medicare Cohort Air Pollution Study, Zeger et al. compared relative mortality risks associated with chronic PM_2.5_ exposure across 250 counties; their results from applying different degrees of spatial smoothing (to adjust for potential spatial confounders) suggest that the evidence for PM_2.5_-mortality association was stronger for larger spatial scale than more local scale comparisons [19]. The decrease in spatially adjusted relative risk with respect to PM is consistent with the findings reported here. Because the broad regional trends in CVD appeared to coincide with PM_2.5_ formation shown by the exposure assessment map, regional adjustment may have over-adjusted the effect estimates for regional scale fine particle pollutants relative to more local scale coarse particle pollutants. Conversely, it might be conjectured that incorporating a state or county-based areal marker should induce greater uncertainty in the PM_10_ effects. However, such local-level adjustment was not adopted as it depends on the usage of arbitrary administrative units which tend not to match PM distribution on a geographical or ecological scale. Consideration also needs to be given to the implications of using aggregate PM exposure data due to the lack of individual-level exposure data. Despite the inclusion of a relatively large set of personal characteristics measures, a strength of this study which helps remove the aggregation effects in analyzing geographically aggregated data, there is still the potential for ecological biases which introduce measurement errors and contribute to uncertainties in effect estimates. As with multilevel studies in general, this limitation needs to be recognized in risk estimation.

In separate analyses to characterize the spatial patterns of the PM effects on CVD, stronger PM-CVD correlations were detected in the eastern-central U.S. (Figure 5). The geographical variations in PM effects may arise from heterogeneity in PM composition across regions, which cannot be adequately captured by concentration-based exposure metrics. A number of studies have reported regional differences in the acute or chronic effects of PM. Using daily time-series data of 1999–2002 on cause-specific hospitalization admissions for 204 U.S. counties, Dominici et al. investigated the short-term effects of PM_2.5_ on cardiovascular and respiratory diseases and noted higher cardiovascular risks in the eastern region (including the Northeast, Southeast, Midwest and the South) [34]. More recently, Zeger et al. evaluated Medicare billing claims for 2000–2005 from urban areas within six miles of a PM_2.5_ monitor; they found significantly elevated mortality risks in the eastern and central regions associated with PM_2.5_ exposure after adjusting for smoking and socioeconomic status, but no PM_2.5_-related effects in the western region [20]. Regarding the adverse effects of PM_2.5_, multiple chemical constituents have been implicated including iron, nickel, zinc, ammonium nitrate, elemental carbon, organic carbon, nitrates, and sulfates [35]–[Bibr pone.0033385-Ostro1] [37]. In a study of the spatial and temporal variations in the PM_2.5_ mixture across 187 U.S. counties for 2000–2005, Bell et al. observed higher sulfate levels in the eastern region, and higher nitrate levels in the western and northern regions [38]. As also noted in the study, a major obstacle to interpreting regional differences in the observed PM effects is that multiple sources of the PM mixture—often in complex interplay—complicate the identification of individual effects of various PM components on a regional or national scale. This suggests that the ability to distinguish and explain the spatial patterns of health risks associated with PM can be improved through localized compositional analyses of ambient PM where definitive source apportionment is feasible.

The present study used kriging for exposure classification, an approach also adopted by the HEI in their reanalysis of the Six Cities and ACS data. Kriging has the advantage of providing unbiased estimates of pollutant levels at unsampled locations with minimum estimated variance, and has been applied with success to model broad scale variations in background air pollution [39]–[Bibr pone.0033385-Lefohn1] [41], where measurements are only made at designated sites. Nonetheless, important constraints on exposure modeling such as limitations in the spatial representativeness of the air sample data used need to be recognized. Because monitoring is costly, the density of monitoring networks is limited. Clustering of monitoring sites is also unavoidable due to monitor placement strategies favoring areas of high pollution levels. The uneven distribution of spatial observations could lead to a low degree of spatial autocorrelation, increased prediction uncertainty and potential exposure misclassification. These considerations suggest that the modeled concentrations should be interpreted as estimated background concentrations, rather than measurements of personal or microenvironmental exposures. In any case, reliance on monitored air pollution data alone provides only a partial picture of the air pollution situation in any area, and supplementing monitored air pollutant measurements with auxiliary factors (e.g. traffic volume, altitude, wind speed/direction, temperature, precipitation) would be worthwhile for more local scale exposure assessment.

Other limitations of the work reported here relate to the cross-sectional nature of the study and the resulting insufficiency of findings to demonstrate a cause-and-effect relationship between the studied air pollutants and CVD morbidity. Also, apart from potential selection biases (e.g. non-coverage of cell phone only households or those with no phone at all) and the restriction of the study population to the selected racial groups, which limit generalizability of results to less-selective populations, a certain degree of inaccuracy in disease outcome and risk factors ascertainment is to be expected with self-reported data; however, it is probable that such inaccuracy would be non-differential, and any bias introduced would only obscure the effects found. In addition, misclassification may arise from exposure assignment according to residency at the time of survey data collection (the BRFSS questionnaires currently do not track migration activities or time spent in the area of residency). Such exposure misclassification is likely to be random and again its main consequence is the attenuation of the effects estimated.

Although the pathomechanisms responsible for the association between air pollution and CVD development or exacerbation have not been fully elucidated, previous observations suggest that exposure to air pollutants elicits morphological changes and systemic inflammatory processes, conditions that may lead to tissue damage and release of bioactive substances into the circulatory system, thus creating direct or indirect insults to the cardiovascular system. Results from air pollution studies show that a large proportion of the urban fine particle mass is made up of primary combustion products from mobile source emissions and includes organic compounds, elemental carbon, and metals [42],[43]. Exposure to many of these toxic pollutant components has been demonstrated as entailing inflammatory and neurogenic responses with local and systemic consequences. Greater toxicity has also been attributed to fine and ultrafine particles (PM with diameter <0.1 µm) due to their high pulmonary deposition efficiency, higher particle number concentration than larger particles and a resulting higher surface area to carry toxic pollutants, as well as their translocation potential [44],[45]. The pathophysiologic consequences arising from PM exposure are both acute and chronic. Short-term exposure to fine particles has been linked to increased risks of myocardial infarction, vasoconstriction, reduced heart rate variability and arrhythmias [46]–[Bibr pone.0033385-Peters2] [48]. The lifetime risks may be influenced by atherosclerotic and inflammatory responses as well as oxidative stress [45],[49],[50]. Importantly, the observed correlations between PM pollution and CVD evidence both acute and protracted mechanisms so a distinction between the short and long-term PM effects cannot be made easily.

While much remains to be discovered about the role of air pollution in cardiovascular pathologic manifestations, this study provides new evidence linking long-term PM exposure to cardiovascular impairment. Indeed, the associations between multiple CVD outcomes and PM remained robust after accounting for major risk factors including demographic characteristics, socioeconomic status, hypertension, hypercholesterolemia, diabetes, smoking, physical activity level and obesity. From a public health perspective, this study underlines the potentiality of air pollution abatement in reducing the morbidity and mortality associated with CVD.

In conclusion, geospatial modeling and multivariate techniques were used to implement a large population assessment of relative cardiovascular risks posed by airborne particulate matter across contiguous U.S.. The findings suggest that improvements in air quality could imply a substantial reduction in the disease burden associated with CVD.

## Supporting Information

Figure S1 **Measured PM concentrations across study sites versus predicted values by the chosen kriging methods.**
(TIF)

Figure S2 **Age-Sex-Race (ASR) adjusted prevalence estimates across study counties—assessed with study samples from the '07 & '09 Behavioral Risk Factor Surveillance System: A—myocardial infarction (MI), B—coronary heart Disease (CHD), and C—stroke (STK).**
(TIF)

Table S1 **Characteristics of study subjects according to quartiles of PM_10_ and PM_2.5_ exposure across study counties.**
(DOC)

Table S2 **Modeled PM_10_ and PM_2.5_ yearly median concentrations (averaging 1999–2005) across study counties by regional strata.**
(DOC)

## References

[1] Tankersley CG, Champion HC, Takimoto E, Gabrielson K, Bedja D (2008). Exposure to inhaled particulate matter impairs cardiac function in senescent mice.. Am J Physiol Regul Integr Comp Physiol.

[2] Cozzi E, Hazarika S, Stallings HW, Cascio WE, Devlin RB (2006). Ultrafine particulate matter exposure augments ischemia-reperfusion injury in mice.. Am J Physiol Heart Circ Physiol.

[3] Chen LC, Nadziejko C (2005). Effects of subchronic exposures to concentrated ambient particles (CAPs) in mice. V. CAPs exacerbate aortic plaque development in hyperlipidemic mice.. Inhal Toxicol.

[4] Sun Q, Wang A, Jin X, Natanzon A, Duquaine D (2005). Long-term air pollution exposure and acceleration of atherosclerosis and vascular inflammation in an animal model.. JAMA.

[5] Burnett RT, Dales R, Krewski D, Vincent R, Dann T (1995). Associations between ambient particulate sulfate and admissions to Ontario hospitals for cardiac and respiratory diseases.. Am J Epidemiol.

[pone.0033385-Morris1] Morris RD, Naumova EN, Munasinghe RL (1995). Ambient air pollution and hospitalization for congestive heart failure among elderly people in seven large US cities.. Am J Public Health.

[pone.0033385-Goldberg1] Goldberg MS, Burnett RT, Bailar JC, Tamblyn R, Ernst P (2001). Identification of persons with cardiorespiratory conditions who are at risk of dying from the acute effects of ambient air particles.. Environ Health Perspect.

[pone.0033385-Peters1] Peters A, Dockery DW, Muller JE, Mittleman MA (2001). Increased particulate air pollution and the triggering of myocardial infarction.. Circulation.

[pone.0033385-Pope1] Pope CA, Dockery DW, Kanner RE, Villegas GM, Schwartz J (1999). Oxygen saturation, pulse rate, and particulate air pollution: a daily time-series panel study.. Am J Respir Crit Care Med.

[pone.0033385-Pope2] Pope CA, Muhlestein JB, May HT, Renlund DG, Anderson JL (2006). Ischemic heart disease events triggered by short-term exposure to fine particulate air pollution.. Circulation.

[pone.0033385-Samet1] Samet JM, Zeger SL, Dominici F, Curriero F, Coursac I (2000). The national morbidity, mortality, and air pollution study. part II: morbidity and mortality from air pollution in the United States.. Res Rep Health Eff Inst.

[pone.0033385-Schwartz1] Schwartz J (1999). Air pollution and hospital admissions for heart disease in eight U.S. counties.. Epidemiology.

[pone.0033385-Schwartz2] Schwartz J, Morris R (1995). Air pollution and hospital admissions for cardiovascular disease in Detroit, Michigan.. Am J Epidemiol.

[14] Zanobetti A, Schwartz J, Dockery DW (2000). Airborne particles are a risk factor for hospital admissions for heart and lung disease.. Environ Health Perspect.

[15] Dockery DW, Pope CA, Xu X, Spengler JD, Ware JH (1993). An association between air pollution and mortality in six US cities.. N Engl J Med.

[16] Pope CA, Thun MJ, Namboodiri MM, Dockery DW, Evans JS (1995). Particulate air pollution as a predictor of mortality in a prospective study of U.S. adults.. Am J Respir Crit Care Med.

[17] Krewski D, Burnett RT, Goldberg MS, Hoover K, Siemiatycki J (2000). Reanalysis of the Harvard Six Cities Study and the American Cancer Society Study on particulate air pollution and mortality.

[pone.0033385-Miller1] Miller KA, Siscovick DS, Sheppard L, Shepherd K, Sullivan JH (2007). Long-term exposure to air pollution and incidence of cardiovascular events in women.. N Engl J Med.

[pone.0033385-Zeger1] Zeger SL, Dominici F, McDermott A, Samet JM (2007). Mortality in the Medicare population and chronic exposure to fine particulate air pollution.. http://www.bepress.com/jhubiostat/paper133/.

[20] Zeger SL, Dominici F, McDermott A, Samet JM (2008). Mortality in the Medicare population and chronic exposure to fine particulate air pollution in urban centers (2000–2005).. Environ Health Perspect.

[21] Centers for Disease Control and Prevention (1998). The Behavioral Risk Factor Surveillance System user's guide.

[pone.0033385-Nelson1] Nelson DE, Holtzman D, Waller M, Leutzinger C, Condon K (1998). Objectives and design of the Behavioral Risk Factor Surveillance System. Proceedings of the section on survey research methods.

[23] Powell-Griner E (1998). Uses and limitations of the Behavioral Risk Factor Surveillance System data. Proceedings of the section on survey research methods.

[24] U.S. Environmental Protection Agency, Technology Transfer Network (TTN) Air Quality System (AQS) website.. http://www.epa.gov/ttn/airs/airsaqs/.

[25] Isaaks EH, Srivastava RM (1989). An introduction to applied geostatistics.

[26] Leenaers H, Okx JP, Burrough PA (1990). Comparison of spatial prediction methods for mapping floodplain soil pollution.. Catena.

[27] Goodman LA (1974). Exploratory latent structure analysis using both identifiable and unidentifiable models.. Biometrika.

[pone.0033385-Formann1] Formann AK (1982). Linear logistic latent class analysis.. Biometrical J.

[pone.0033385-Clogg1] Clogg CC, Goodman LA (1984). Latent structure analysis of a set of multi-dimensional contingency tables.. J Amer Statist Assoc.

[30] Dayton CM, Macready GB (1988). Concomitant-variable latent class models.. J Amer Statist Assoc.

[31] Agresti A, Booth JG, Hobert JP, Caffo BS (2000). Random effects modeling of categorical response data.. Sociol Methodol.

[pone.0033385-Vermunt1] Vermunt JK, Van Dijk L (2001). A nonparametric random-coefficients approach: the latent class regression model.. Multilevel Modelling Newsletter.

[33] Agresti A (2002). Categorical data analysis.

[34] Dominici F, Peng RD, Bell ML, Pham L, McDermott A (2006). Fine particulate air pollution and hospital admission for cardiovascular and respiratory diseases.. JAMA.

[35] Burnett RT, Brook J, Dann T, Delocla C, Philips O (2000). Association between particulate- and gas-phase components of urban air pollution and daily mortality in eight Canadian cities.. Inhal Toxicol.

[pone.0033385-Ostro1] Ostro B (1995). Fine particulate air pollution and mortality in two southern California counties.. Environ Res.

[37] Ostro B, Feng WY, Broadwin R, Green S, Lipsett M (2007). The effects of components of fine particulate air pollution on mortality in California: results from CALFINE.. Environ Health Perspect.

[38] Bell ML, Dominici F, Ebisu K, Zeger SL, Samet JM (2007). Spatial and temporal variation in PM_2.5_ chemical composition in the United States for health effects studies.. Environ Health Perspect.

[39] Beelen R, Hoeka G, Pebesma E, Vienneau D, de Hoogh K (2009). Mapping of background air pollution at a fine spatial scale across the European Union.. Sci Total Environ.

[pone.0033385-Lefohn1] Lefohn A, Knudsen HP, McEvoy LR (1988). The use of kriging to estimate monthly ozone exposure parameters for the southeastern United States.. Environ Pollut.

[41] Liao D, Peuquet DJ, Duan Y, Whitsel EA, Dou J (2006). GIS approaches for the estimation of residential-level ambient PM concentrations.. Environ Health Perspect.

[42] Health Effects Institute (2002). Understanding the health effects of components of the particulate matter mix: progress and next steps.

[43] Kim S, Shen S, Sioutas C (2002). Size distribution and diurnal and seasonal trends of ultrafine particles in source and receptor sites of the Los Angeles basin.. J Air Waste Manage Assoc.

[44] Oberdörster G (2001). Pulmonary effects of inhaled ultrafine particles.. Int Arch Occup Environ Health.

[45] Polichetti G, Cocco S, Spinali A, Trimarco V, Nunziata A (2009). Effects of particulate matter (PM_10_, PM_2.5_ and PM_1_) on the cardiovascular system.. Toxicology.

[46] Brook RD, Brook JR, Urch B, Vincent R, Rajagopalan S (2002). Inhalation of fine particulate air pollution and ozone causes acute arterial vasoconstriction in healthy adults.. Circulation.

[pone.0033385-Peters2] Peters A, Lui E, Verrier RL, Schwartz J, Gold DR (2000). Air pollution and incidence of cardiac arrhythmia.. Epidemiology.

[48] Pope CA, Eatough DJ, Gold DR, Pang Y, Nielsen KR (2001). Acute exposure to environmental tobacco smoke and heart rate variability.. Environ Health Perspect.

[49] Delfino RJ, Sioutas C, Malik S (2005). Potential role of ultrafine particles in associations between airborne particle mass and cardiovascular health.. Environ Health Perspect.

[50] Yang W, Omaye ST (2009). Air pollutants, oxidative stress and human health.. Mutat Res.

