# Supplementary figures and images for: Effects of Particulate Air Pollution on Cardiovascular Health: A Population Health Risk Assessment
Source: PLoS One. 2012 Mar 14;7(3):e33385. doi: 10.1371/journal.pone.0033385 (PMC3303831; doi:10.1371/journal.pone.0033385)

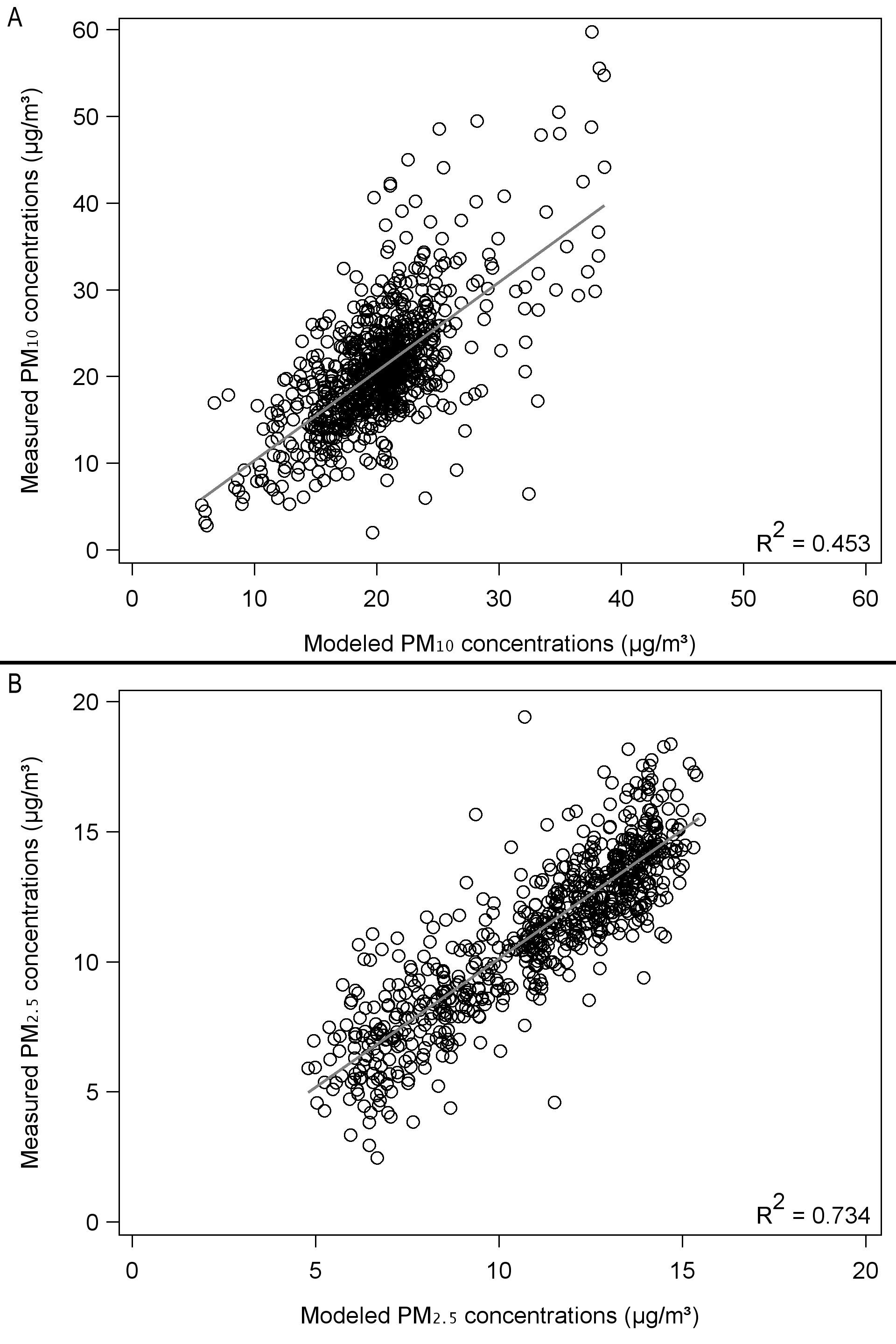

Supplement: Figure S1 — Measured PM concentrations across study sites versus predicted values by the chosen kriging methods. (TIF) [file pone.0033385.s001.tif]

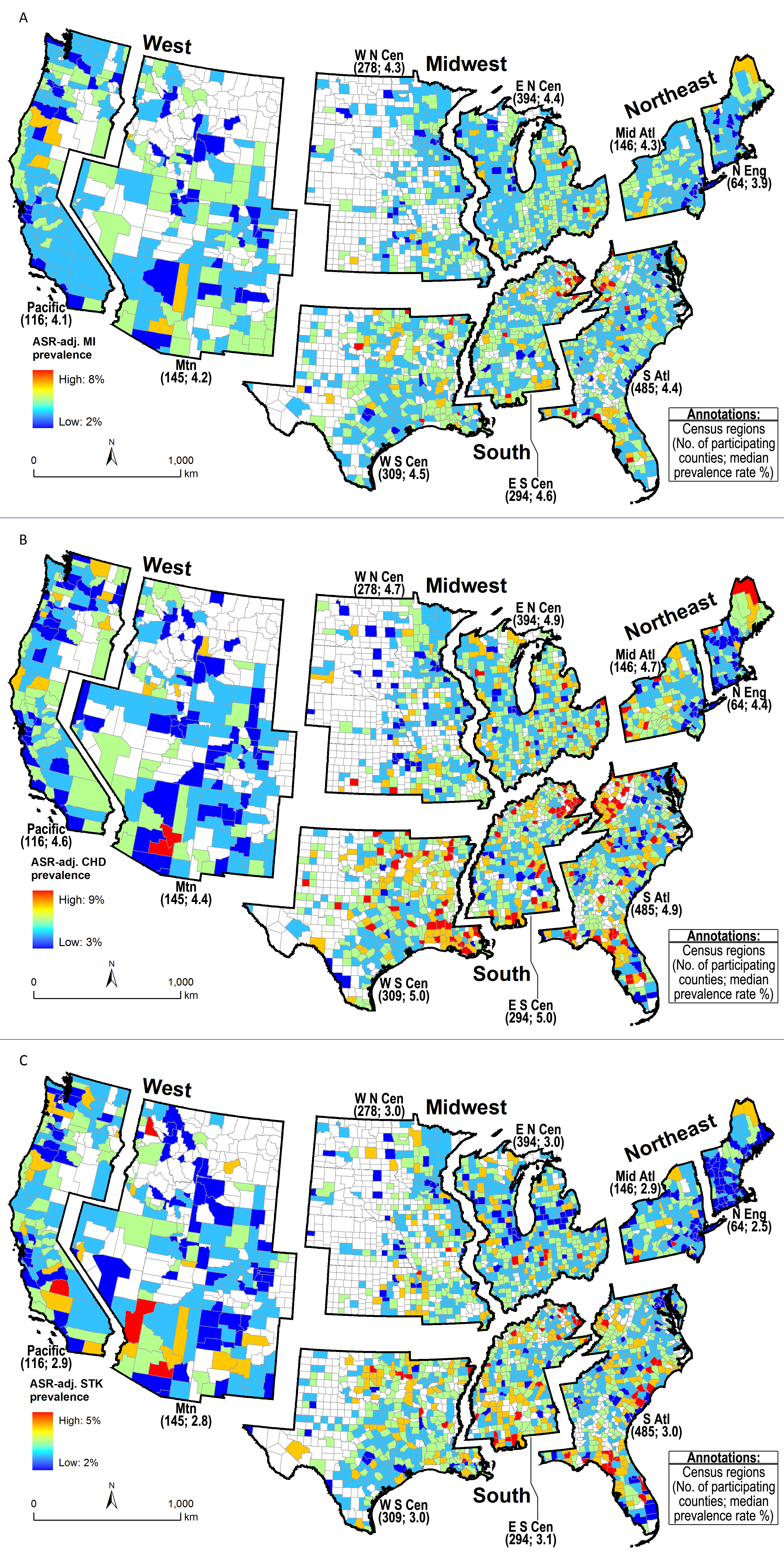

Supplement: Figure S2 — Age-Sex-Race (ASR) adjusted prevalence estimates across study counties—assessed with study samples from the '07 & '09 Behavioral Risk Factor Surveillance System: A—myocardial infarction (MI), B—coronary heart Disease (CHD), and C—stroke (STK). (TIF) [file pone.0033385.s002.tif]
