# Supplementary material for: Effects of Particulate Air Pollution on Cardiovascular Health: A Population Health Risk Assessment
Source: PLoS One. 2012 Mar 14;7(3):e33385. doi: 10.1371/journal.pone.0033385 (PMC3303831; doi:10.1371/journal.pone.0033385)
Supplement: Table S1 — Characteristics of study subjects according to quartiles of PM10 and PM2.5 exposure across study counties. (DOC) [file pone.0033385.s003.doc]

| *Characteristic* | ***PM_10_ quartile (µg/m^3^)*** | | | | ***PM_2.5_ quartile (µg/m^3^)*** | | | |
| --- | --- | --- | --- | --- | --- | --- | --- | --- |
|  | *6.73-18.38* | *18.39-20.17* | *20.18-21.21* | *21.22-33.96* | *4.68-9.14* | *9.15-11.37* | *11.38-12.83* | *12.84-15.56* |
| *N* (unweighted)* | 107,302 | 84,571 | 85,511 | 223,331 | 185,846 | 147,270 | 91,444 | 76,155 |
| *Age group (%)* |  |  |  |  |  |  |  |  |
| 18-24 | 1.2 | 1.2 | 1.4 | 1.4 | 1.2 | 1.3 | 1.5 | 1.4 |
| 25-34 | 6.8 | 7.0 | 7.7 | 7.6 | 6.9 | 7.4 | 8.0 | 7.5 |
| 35-44 | 14.7 | 14.8 | 15.0 | 15.0 | 14.4 | 15.1 | 15.6 | 14.8 |
| 45-54 | 23.0 | 21.9 | 22.0 | 21.9 | 22.3 | 22.1 | 21.9 | 22.1 |
| 55-64 | 24.2 | 23.9 | 23.9 | 23.7 | 24.5 | 23.4 | 23.4 | 23.9 |
| 65+ | 30.1 | 31.2 | 30.0 | 30.5 | 30.7 | 30.7 | 29.6 | 30.3 |
| *Male (%)* | 40.5 | 39.6 | 39.2 | 38.6 | 40.6 | 39.1 | 38.2 | 37.6 |
| *Race/Ethnicity (%)* |  |  |  |  |  |  |  |  |
| White, non-Hispanic | 93.2 | 88.8 | 84.7 | 83.1 | 91.3 | 87.6 | 76.0 | 85.2 |
| Black, non-Hispanic | 2.4 | 5.7 | 10.9 | 11.1 | 1.4 | 7.4 | 20.1 | 12.6 |
| Hispanic | 4.5 | 5.5 | 4.4 | 5.8 | 7.3 | 5.0 | 3.9 | 2.3 |
| *Education (%)* |  |  |  |  |  |  |  |  |
| Less than H.S. diploma | 5.6 | 6.7 | 7.5 | 8.1 | 5.6 | 7.1 | 8.7 | 9.8 |
| H.S. diploma or G.E.D. | 27.1 | 26.5 | 27.5 | 28.7 | 25.7 | 28.1 | 28.1 | 31.8 |
| Some post-H.S. | 26.2 | 26.9 | 28.3 | 26.8 | 28.9 | 26.4 | 25.0 | 25.5 |
| College degree or higher | 41.1 | 39.8 | 36.8 | 36.4 | 39.8 | 38.4 | 38.3 | 32.9 |
| *Household income per year (%)* |  |  |  |  |  |  |  |  |
| Less than $15,000 | 8.3 | 8.6 | 9.2 | 10.2 | 7.9 | 9.1 | 10.7 | 11.9 |
| $15,000 to 24,999 | 14.7 | 15.6 | 15.9 | 16.4 | 15.0 | 15.6 | 15.8 | 18.3 |
| $25,000 to 34,999 | 11.6 | 11.7 | 11.9 | 12.0 | 11.9 | 11.7 | 11.3 | 12.7 |
| $35,000 to 49,999 | 16.0 | 15.9 | 15.9 | 15.5 | 16.7 | 15.4 | 14.6 | 15.6 |
| $50,000 or more | 49.4 | 48.1 | 47.2 | 45.9 | 48.6 | 48.2 | 47.5 | 41.5 |
| *Married (%)* | 59.2 | 58.1 | 59.6 | 57.7 | 61.0 | 57.6 | 55.4 | 57.1 |
| *Smoking history (%)* |  |  |  |  |  |  |  |  |
| Current smoker† | 14.8 | 15.3 | 16.0 | 16.6 | 14.1 | 16.4 | 16.7 | 18.5 |
| Former smoker†† | 35.4 | 33.0 | 30.7 | 30.8 | 33.1 | 32.9 | 30.6 | 30.3 |
| Never smoked | 49.8 | 51.7 | 53.3 | 52.6 | 52.8 | 50.7 | 52.8 | 51.2 |
| *Leisure time exercise: none in past 30 days (%)*# | 21.8 | 22.7 | 24.7 | 26.0 | 20.9 | 25.0 | 26.7 | 28.4 |
| *Alcohol use: total no. of drinks in past 30 days (mean±SE)* | 12.4±0.09 | 11.6±0.12 | 10.0±0.11 | 9.5±0.06 | 11.6±0.07 | 11.1±0.09 | 9.6±0.10 | 8.2±0.10 |
| *Body Mass Index (mean±SE)* | 27.5±0.02 | 27.7±0.02 | 28.1±0.02 | 28.0±0.01 | 27.6±0.01 | 27.9±0.02 | 28.2±0.02 | 28.2±0.02 |
| *Hypertension (%)* | 36.7 | 38.5 | 41.1 | 40.8 | 37.0 | 39.6 | 42.0 | 42.8 |
| *Hypercholesterolemia (%)* | 42.0 | 42.4 | 43.4 | 43.7 | 42.1 | 43.2 | 43.8 | 44.2 |
| *Diabetes (excluding gestational diabetes) (%)* | 10.5 | 11.7 | 12.6 | 13.2 | 10.7 | 12.3 | 13.5 | 14.3 |
| *Cardiovascular complications including MI, CHD or STK (%)* | 11.0 | 11.7 | 12.4 | 12.5 | 11.0 | 12.2 | 12.4 | 13.5 |

* The unweighted BRFSS 2007 and 2009 samples were restricted to non-Hispanic whites/blacks and Hispanics with identified disease state pertaining to history of myocardial infarction (MI), coronary heart disease (CHD) and stroke (STK). Excluding incomplete respondents (those on whom at least one covariate or outcome measure had missing data), 494,358 participants were available for the LCR analysis, and the sample sizes for MI, CHD and STK risk assessments were 498,815, 496,909, and 499,667 respectively. Taken together, a total of 500,715 respondents were included in the data tabulation.

† Respondents that reported having smoked at least 100 cigarettes (5 packs) in their lifetime and currently smoke some days or every day.

†† Respondents that reported having smoked at least 100 cigarettes in their lifetime and currently do not smoke.

# Leisure time exercise defined as doing physical activity or exercise during the past 30 days other than one’s regular job.
