# Supplementary material for: Effects of Particulate Air Pollution on Cardiovascular Health: A Population Health Risk Assessment
Source: PLoS One. 2012 Mar 14;7(3):e33385. doi: 10.1371/journal.pone.0033385 (PMC3303831; doi:10.1371/journal.pone.0033385)
Supplement: Table S2 — Modeled PM10 and PM2.5 yearly median concentrations (averaging 1999–2005) across study counties by regional strata. (DOC) [file pone.0033385.s004.doc]

| *Regional* | *No. of counties* | *PM_10_ concentrations (µg/m^3^)* | | | | *PM_2.5_ concentrations (µg/m^3^)* | | | |
| --- | --- | --- | --- | --- | --- | --- | --- | --- | --- |
| *strata* | *(No. of subjects)* | Mean (SD) | Median | Min | Max | Mean (SD) | Median | Min | Max |
| *Midwest* | 672 (101,575) |  |  |  |  |  |  |  |  |
| E N Cen | 394 (47,988) | 19.35 (2.65) | 20.20 | 9.57 | 25.46 | 11.88 (2.21) | 12.54 | 5.67 | 14.97 |
| W N Cen | 278 (53,587) | 20.42 (2.78) | 20.78 | 7.79 | 25.48 | 9.66 (1.72) | 9.91 | 5.05 | 13.55 |
| *Northeast* | 210 (106,960) |  |  |  |  |  |  |  |  |
| Mid Atl | 146 (36,023) | 17.59 (2.97) | 18.66 | 10.04 | 22.56 | 10.84 (1.50) | 10.87 | 7.57 | 13.86 |
| N Eng | 64 (70,937) | 14.41 (2.71) | 13.70 | 10.70 | 20.23 | 8.63 (1.03) | 8.19 | 7.30 | 10.68 |
| *South* | 1,088 (178,176) |  |  |  |  |  |  |  |  |
| E S Cen | 294 (33,816) | 20.54 (1.71) | 20.37 | 15.51 | 24.99 | 12.96 (0.85) | 13.22 | 10.91 | 14.63 |
| S Atl | 485 (105,902) | 19.99 (1.94) | 20.39 | 14.17 | 23.62 | 12.49 (1.42) | 12.58 | 8.02 | 15.56 |
| W S Cen | 309 (38,458) | 20.42 (0.91) | 20.66 | 17.02 | 22.30 | 10.16 (1.31) | 10.18 | 6.81 | 12.46 |
| *West* | 261 (114,004) |  |  |  |  |  |  |  |  |
| Mtn | 145 (63,743) | 18.62 (4.30) | 19.31 | 6.73 | 33.96 | 6.43 (0.79) | 6.47 | 4.68 | 8.90 |
| Pacific | 116 (50,261) | 17.70 (3.09) | 17.39 | 10.67 | 32.39 | 7.40 (1.27) | 7.27 | 5.48 | 13.12 |
|  |  |  |  |  |  |  |  |  |  |
